# Editorial: One Health and Veterinary Regenerative Medicine: Translational Applications

**DOI:** 10.3389/fvets.2022.959564

**Published:** 2022-07-05

**Authors:** Tracy L. Webb, Jan H. Spaas, Debbie J. Guest

**Affiliations:** ^1^Department of Clinical Sciences, Colorado State University, Fort Collins, CO, United States; ^2^Boehringer-Ingelheim Animal Health USA, Athens, GA, United States; ^3^Department of Morphology, Imaging, Orthopedics, Rehabilitation and Nutrition, Faculty of Veterinary Medicine, Ghent University, Merelbeke, Belgium; ^4^Department of Clinical Science and Services, Royal Veterinary College, London, United Kingdom

**Keywords:** regenerative medicine, one health, translational, veterinary, clinical studies, naturally occurring

Regenerative Medicine has been heralded as having the potential to provide much needed treatments for many significant diseases in human and veterinary medicine (1). However, the restorative/regenerative potential for these therapies has yet to be fully achieved. Recent years and the current pandemic have brought attention to some challenges in regenerative medicine as well as the benefits of interdisciplinary collaborations and One Health approaches that recognize the close connection between the health of people and animals. Veterinary medicine and naturally occurring diseases in animals can provide impactful information and help direct and refine new treatments and prevention strategies for the betterment of both human and animal health and the environment. A thorough assessment of the current strengths, gaps, and priorities in veterinary and human regenerative medicine can help identify critical steps to accelerate the progress and impact of regenerative therapies.

With this Research Topic, we seek to highlight the current state of veterinary regenerative medicine, with an emphasis on One Health and translation of novel therapies to the clinic, as a means of encouraging collaboration and a call to action on areas of need. We identified five focus areas for the Research Topic: (1) regulatory topics, differences, and hurdles; (2) safety and efficacy issues; (3) challenges in veterinary clinical studies, including study quality and ethical discussions; (4) current naturally occurring animal models; and (5) sustainability in regenerative medicine.

Regulation of regenerative medicine, with its many novel and diverse products, has been an important and evolving area of discussion (2). Getting safe and effective regenerative therapies tested, manufactured, and to market involves development of new collaborations, terminology, protocols, and procedures and close attention to detail. Specific topics range from the current state of regulation in veterinary regenerative medicine, including comparisons between veterinary species, human regenerative medicine, and regulatory agencies, to advocating for transparency in study methods and sound, well-powered experimental designs with appropriate control groups, blinding, and randomization. One of the six papers included in this Research Topic discusses challenges and recommendations in manufacturing mesenchymal stromal cells (MSC) for the treatment of osteoarthritis in canine patients. Ivanovska et al. highlight critical issues to be addressed in MSC products for veterinary use. They provide recommendations based on standards and strategies applied in human MSC manufacturing to facilitate the development of quality standards that will contribute to the standardization of cell manufacturing methods and improved quality assurance across the field.

Involving both preclinical and clinical research, ethical considerations in the dynamic arena of regenerative medicine are complex and important to iteratively address (3). Regenerative medicine, similar to transfusion and transplant medicine, must address the rights of both donors and recipients. Aligned with regulatory agencies and the more complex review process for these products, many groups have highlighted the risks associated with use of promising, but currently unapproved, therapies to address the significant unmet therapeutic needs of both veterinary and human patients. Accordingly, transparent, effective communication strategies need to be directed toward all audiences, covering areas such as study recruitment and consenting practices, regulatory requirements, strengths and limitations of using companion animals as human models or in One Health approaches, use of therapies with limited evidence of efficacy, among others.

Thoughtful use of current experimentally induced and naturally occurring animal models remains essential for investigating product safety, mechanism of action, and efficacy in the target species or predicting efficacy in similar disease conditions in other species. Two papers in the Research Topic look at experimentally induced models to provide initial proof of concept data to support further study. Xu et al. evaluates the impact of accelerated high frequency repetitive transcranial magnetic stimulation (aHF-rTMS) on healthy beagle metabolism finding that, similar to human psychiatric disorders, active aHF-rTMS modifies glucose metabolism and therefore may be a valid treatment option for mentally disordered dogs. Depuydt et al. present two studies showing that, in greater than 92% of the treated horses, administration of allogeneic, tenogenic primed MSCs did not induce a cellular or humoral immune response following a single or repeated intralesional treatment in horses with tendon injury. The first study was in experimentally induced tendon injury, informing the second study in horses with naturally occurring injury. Larger field studies are needed to confirm these initial findings and support the safe and effective use of these therapeutics.

The three remaining papers included in this Research Topic discuss the translational impacts of naturally occurring disease models. In Arzi et al. the authors assert that experience gained through use of cell therapies in companion animals with naturally occurring disease represents a unique and under-utilized resource that could serve as a critical bridge between laboratory and preclinical models and successful human clinical trials through a One-Health approach. Willemsen et al. look more specifically at hip dysplasia demonstrating that conservative treatment and surgical interventions for the disease are very similar for both dogs and humans, and therefore future integration of knowledge and experiences could be beneficial for both species. A case report by Melotti et al. presents results suggesting positive synergic effects of a repeated intralesional injection of adipose derived-MSCs and platelet rich plasma for treatment of a horse with chronic tendonitis, a disease shared by several species, with long-term effects and improvement in quality of life and athletic performance.

Perhaps identifying a significant gap in current regenerative medicine practices and priorities, no papers were submitted addressing sustainability. This gap has similarly been identified across the veterinary medical field and is an area of need and opportunity for meaningful impact (4, 5). Current novel regenerative therapies are frequently very resource intensive, using large amounts of expensive and non-recyclable raw materials. Future research should focus on novel ways to decrease the use of non-reusable consumables and other resources in order to decrease the current financial and environmental footprint of regenerative medicine and thereby increase the potential of the field to positively impact patient outcomes.

Although a relatively small Research Topic, One Health and Veterinary Regenerative Medicine: Translational Applications contributes to the advancement of the field both in identifying current priorities and research focus areas as well as defining significant gaps, challenges, and areas for advancement. Hopefully these and similar efforts will move the field closer to realizing the full potential of regenerative medicine for veterinary and human patients.

## Author Contributions

All authors listed have made a substantial, direct, and intellectual contribution to the work and approved it for publication.

## Conflict of Interest

JS declares to be employed by Boehringer-Ingelheim owning several products based on mesenchymal stem cells and an inventor of the related patents. The remaining authors declare that the research was conducted in the absence of any commercial or financial relationships that could be construed as a potential conflict of interest.

## Publisher's Note

All claims expressed in this article are solely those of the authors and do not necessarily represent those of their affiliated organizations, or those of the publisher, the editors and the reviewers. Any product that may be evaluated in this article, or claim that may be made by its manufacturer, is not guaranteed or endorsed by the publisher.
